# Inflammatory myofibroblastic tumor with extensive involvement of the bladder in an adolescent: a case report

**DOI:** 10.1186/1477-7819-11-206

**Published:** 2013-08-19

**Authors:** Xin Lin Yi, Hao Yuan Lu, Yue Xian Wu, Wen Hui Li, Qing Gui Meng, Ji Weng Cheng, Yong Tang, Yu Liu, Xian Zhong Bai

**Affiliations:** 1 Department of Urology, Cancer Hospital of Guangxi Medical University & Guangxi Cancer Research Institute, Nanning 530021, People’s Republic of China; 2 Department of Respiratory Diseases, Nanfang Hospital, Southern Medical University, Guangzhou 510515, People’s Republic of China; 3 Department of Surgery, Gongchang Hospital of HuBei Province, Jiangli 433301, People’s Republic of China; 4 Department of Urology, Guangxi National Hospital, Nanning 530000, People’s Republic of China

**Keywords:** Bladder, Inflammatory myofibroblastic tumor

## Abstract

Inflammatory myofibroblastic tumor (IMT) is a rare lesion of unclear pathogenesis that shows a wide, highly variable spectrum of clinical behavior. We describe the case of a 17-year-old boy with a large IMT that infiltrated the bladder, ileocecal junction, peritoneum and pelvic retroperitoneal space. The tumor was associated with extensive toughening and thickening of the bladder, and, although it showed a tendency for invasive growth, it affected mainly the bladder and adjacent tissue. To the best of our knowledge, this case report is the first to describe an IMT involving the entire bladder and several adjacent pelviabdominal organs. The bladder wall was tough and could hardly be cut by scalpel. Levels of inflammatory response markers such as C-reactive protein fell after surgery.

## Background

Inflammatory myofibroblastic tumor (IMT) is a rare lesion of unclear pathogenesis that exhibits highly variable and unpredictable clinical behavior [1]. Developing a clear understanding of IMT pathophysiology is challenging, in part because of variability in terminology. For instance, IMTs are now classified as neoplastic lesions, but they were termed *inflammatory pseudotumors* 20 years ago [2].

IMTs in adults usually occur in the lungs [3]. Abdominal and retroperitoneal IMTs, by comparison, are usually larger and more aggressive and show high local recurrence rates [4]. In rare cases, IMTs have been found in the bladder, and some authors have suggested that these tumors are sufficiently different from other IMTs that they should be considered a special category [5].

Herein we present the case of a 17-year-old boy with a large IMT that encroached on the bladder, ileocecal junction, peritoneum and pelvic retroperitoneal space. To the best of our knowledge, this case report is the first to describe an IMT involving the entire bladder and several adjacent pelviabdominal organs.

## Case presentation

A 17-year-old boy was referred to our hospital because of urinary frequency, urinary urgency and weight loss over the preceding 2 months. He had no history of urinary tract infection or abdominal trauma, nor had he ever undergone surgery. A physical examination showed him to be of thin build and pale. A tender, fixed mass in the suprapubic area was found by palpation. Laboratory test results showed elevated levels of numerous disease indicators, including the inflammatory marker C-reactive protein (CRP) and globins (Table 1).

**Table 1:** **Laboratory test results**^**a**^

**Test**	**At admission**	**50 days after surgery**	**Normal reference range**
Blood			
C-reactive protein	89.05 mg/L	10.16 mg/L	0 to 10 mg/L
hs-CRP	6.4 mg/L	3 mg/L	0 to 3 mg/L
IgG	27.81 g/L	19.9 g/L	8 to 16 g/L
Platelets	386 × 10^9^/L	315 × 10^9^/L	100 to 300 × 10^9^/L
Total protein	96.7 g/L	ND	60 to 80 g/L
Globulin	54.8 g/L	ND	25 to 35 g/L
Albumin/globulin ratio	0.76	ND	1 to 2.5
White blood cell count	9.18 × 10^9^/L	6 × 10^9^/L	3.97 to 9.15 × 10^9^/L
Percentage of neutrophils	74.6%	62.3%	45 to 77%
Percentage of lymphocytes	19.0%	27.8%	20 to 40%
Hemoglobin	119 g/L	98.0 g/L	131 to 172 g/L
Mean corpuscular volume (MCV)	77.90 fl	81.00 fl	86 to 100 fl
Mean corpuscular hemoglobin (MCH)	25.80 pg	25.20 pg	26 to 31 pg
Alexin C4	0.41 g/L	0.32 g/L	0.2 to 0.4 g/L
Alexin C3	1.47 g/L	1.52 g/L	0.9 to 1.5 g/L
Erythrocyte sedimentation rate	ND	55 mm/h	0 to 15 mm/h
Urine			
Urinary microalbumin	≥150 mg/L	ND	<20 mg/L

^a^*hs-CRP* high-sensitivity C-reactive protein, *IgG* immunoglobulin G, *ND* not done.

The patient was negative for the following tests and markers: colonoscopy, thyroid hormones, hepatitis B and C infection, IgM, IgA, antistreptolysin O prostate-specific antigen and α-fetoprotein (AFP). The patient was also negative for the following tumor makers: carcinoembryonic antigen (CEA), CA 125, CA 15–3, CA 19–9, CA 242 and CA 72–4.

**Figure 1:**
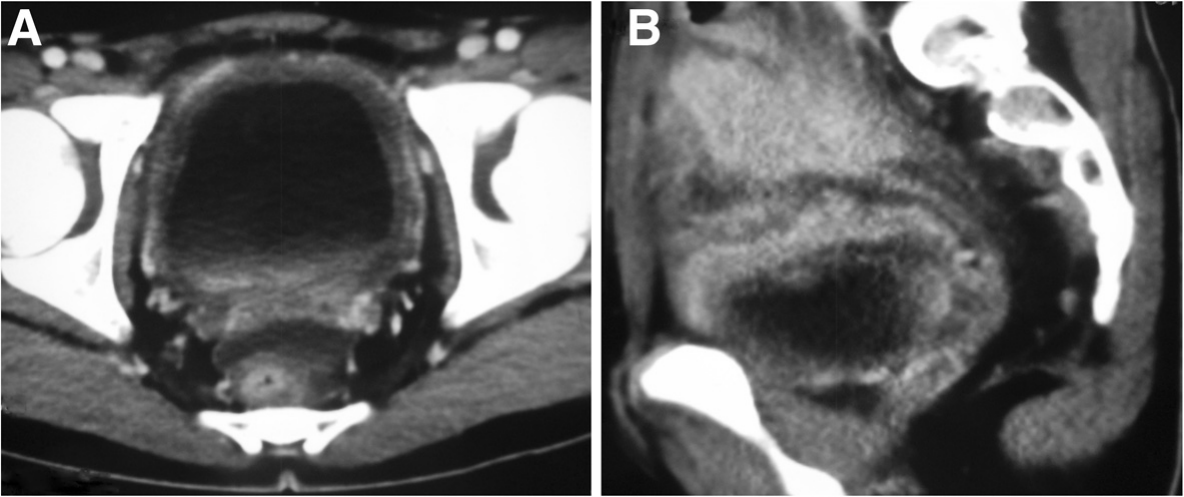
**Enhanced computed tomography scan of the pelvis. (A)** Enhanced computed tomography (CT) scan of the pelvis showing uneven thickening of the bladder wall and heterogeneous enhancement performance. **(B)** Enhanced CT revealing infiltration by a large, irregular, neoformative mass involving the adjacent peritoneum and ileocecal junction.

Contrast-enhanced computed tomography (CT) revealed a 5 cm × 6 cm × 7–cm, irregular, neoformative mass involving the ileocecal junction, bladder and adjacent peritoneum. The bladder wall showed uneven thickening and heterogeneous enhancement performance (Figure 1). Some ascites were present.

**Figure 2:**
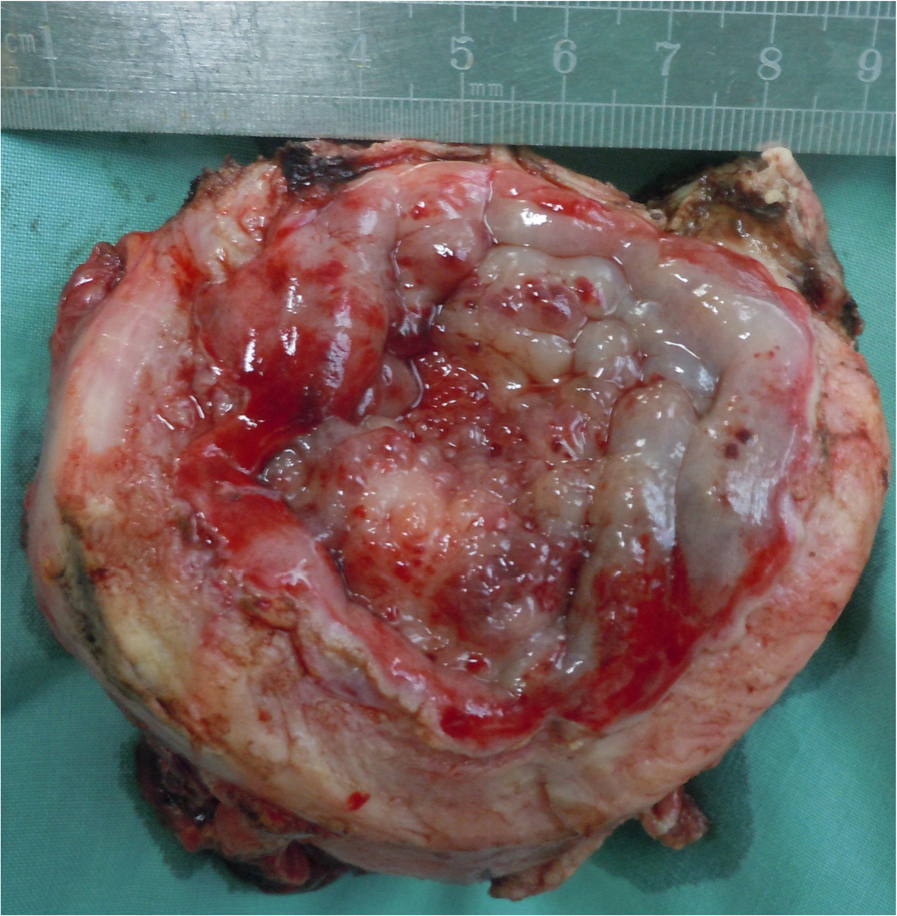
**Resected specimen.** Macrobiopsy (8 cm × 6 cm × 4 cm) obtained during exploratory laparotomy showing the bladder with a white, fibrotic-like, tough wall and a polished mucosa.

Exploratory laparotomy was performed. A large, poorly defined, noncapsulated, fixed mass was found involving the inferior ventrimeson peritoneum, ileocecal junction, mesentery, bladder and perivesical fat tissue. The bladder wall was tough and difficult to cut, reminiscent of coconut endocarp or thick eggshell. It showed extensive, uneven thickening (Figure 2). Its thickness was at least 0.9 cm and significantly more in some areas. Extensive pelvic retroperitoneal fibrosis was observed It extended into the seminal vesicles and Denonvilliers’ fascia and encased the inferior ureter, vas deferens.

Surgery was performed to excise the tumor. The peritoneum and ileocecal junction were partially resected, the bladder was also partially resected and ileocystoplasty was performed.

**Figure 3:**
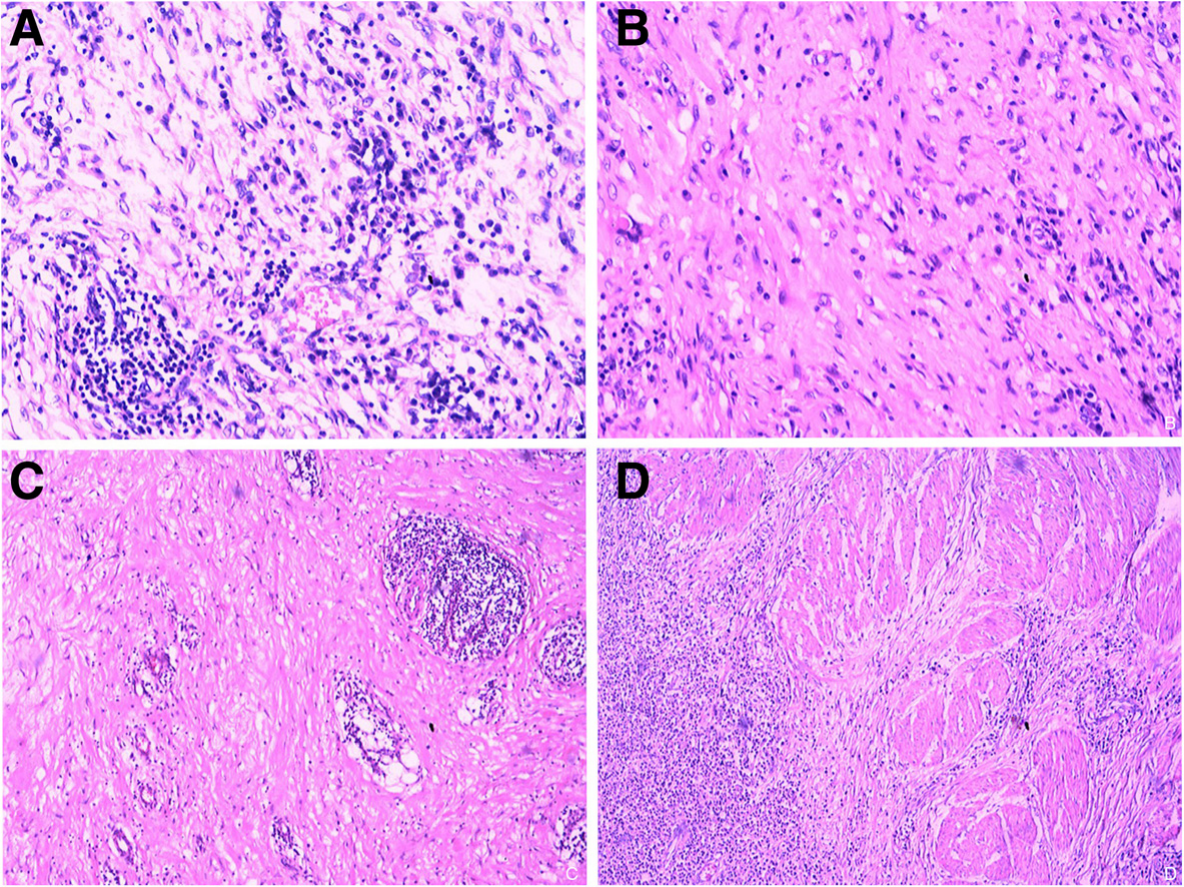
**Lesion characteristics under microscope. (A)**, and **(B)** High-power magnification photomicrographs of the bladder showing proliferating myofibroblasts and fibrous tissue of full thickness. Large numbers of lymphocytes, plasmocytes and neutrophils infiltrated the plasma of the bladder, leading to microabscesses **(C)**. **(D)** Detrusor muscles of the bladder were not collapsed.

Intraoperative microscopic analysis suggested a spindle cell lesion with mucoid degeneration. Subsequent pathology analysis showed bland, spindle-shaped myofibroblasts loosely arranged in a myxoid stroma with plasma cells and inflammatory cells (Figure 3). Resected bladder tissue specimens were positive for smooth muscle actin and CD68 and negative for desmin and anaplastic lymphoma kinase (ALK). They showed low expression of Ki-67. Mesenteric lymph nodes (three of three) showed reactive inflammation. A definitive diagnosis of IMT of the bladder and colon was made.

Laboratory tests showed substantial reductions in numerous markers of disease and inflammation by 50 days after surgery (Table 1). Follow-up at 6 months showed no evidence of tumor recurrence, and ultrasound and cystoscopy tests gave negative results.

## Discussion

IMTs range from benign to locally invasive [1], and they have been proposed to be caused by chronic infection, an immune or autoimmune condition, trauma, surgery or other malignancies [5]. The primary tumor is usually found as an isolated nodular lesion in the lungs, abdomen or retroperitoneum in adults [3,4] and intraabdominally in the mesentery and omentum in children [6]. They rarely occur in the genitourinary tract. IMTs in the lower urogenital tract are probably a special type of IMT. They are often associated with surgical trauma and have been proposed to be an exuberant reparative reaction [7]. We were unable to determine a likely cause of the IMT in our patient. IMTs in the bladder can be associated with von Recklinghausen disease [8], but the characteristic orbital pseudotumor and thyroid lesions were absent in our patient.

Although some IMTs are very aggressive, spreading locally and recurring after successful excision [9], the IMT lesions in our patient appeared to be inflammatory rather than malignant. This assessment is based on several lines of anatomical and biochemical evidence. First, the detrusor muscles of the bladder were intact, whereas they are usually damaged when a bladder tumor is aggressively malignant (Figure 3). Second, the patient presented with elevated levels of several inflammatory markers: serum CRP, high-sensivity CRP, white cells and immunoglobulin G. These levels declined with disease remission, as observed previously [10]. Third, the patient was negative for AFP and CEA- and CA-type tumor markers.

Histologically, IMTs are characterized by atypical spindle-cell proliferation in a myxoid to collagenous stroma with a prominent inflammatory infiltrate comprised primarily of plasma cells and lymphocytes [11]. The IMTs may be mistaken for spindle-cell sarcoma, sarcomatoid carcinoma, rhabdomyosarcoma or leiomyosarcoma [6].

It is important to avoid mistaking sarcomatoid carcinoma for IMT. The former, with its prominent myxoid and sclerosing stroma, may be mistaken for IMT [12]. However, IMTs lack severe cytologic atypia, atypical mitotic figures and extensive necrosis. In addition, IMT often features a myxoid background, inflammatory infiltrate and prominent, slitlike vessels [12]. Most of all, the detrusor muscles of the bladder in IMTs were not collapsed, as our current case shows. The presence of necrosis at the tumor–detrusor muscle interface may help to distinguished sarcoma from IMT [11].

The IMT in our patient was negative for ALK-1, which has been reported in up to 87.5% of IMTs, whereas ALK-1 was found to be negative in sarcomatoid carcinoma, leiomyosarcoma and neurofibroma [13], but such reactivity does not appear to correlate with the prognosis of IMTs. The IMT in our patient involved several organs, which is uncommon. So many tissues were involved in our patient, in fact, that we could not identify the primary site. We are confident that the tumor did not originate in the intestine because the intestinal tissue adjacent to the bowel appeared normal, and it did not show adhesions to the bowel. Lesions of the urachus can infiltrate into the rectus abdominis muscle, colon, bladder and terminal ileum [14], but our patient had a normal umbilicus. Thus, although the lesion in our patient showed a tendency for invasive growth, it affected mainly adjacent fixed tissue.

IMTs in the bladder have been found at numerous sites [15], but they are found most often at the dome [6]. In our patient, however, the trigone of the bladder was not involved, except for secondary invasion from the posterior wall. The bladder wall was tough and, except for part of the trigone, could hardly be cut by scalpel. The mucosa was thickening with waxy luster. Interestingly, although the bladder trigone was thickened in our patient, it was soft and had a normal appearance.

IMTs in the bladder usually appear as polypoid intraluminal or submucosal masses with or without extension into the perivesical fat [16]. Our patient, in contrast, showed a tough, diffuse lesion featuring uneven thickening of the bladder. To the best of our knowledge, this case report is the first to describe such an IMT.

The radiological characteristics of the IMT of our patient, like those of most IMTs, were between those of malignant tumors and those of inflamed tissue. As in the case of our patient, CT typically shows a solitary, uneven, peripheral, sharply circumscribed mass, as well as thickening of the bladder wall. Enhanced CT showed heterogeneous performance.

IMTs have been successfully treated with steroids, nonsteroidal inflammatory drugs, radiotherapy and chemotherapy [17]. Nevertheless, the treatment of choice for IMT remains complete surgical excision. A review of 35 pediatric patients with IMT (median age 7 yr) who were treated by surgical excision found no evidence of recurrence or metastasis after a median follow-up of 1.5 years among 29 of the patients [6].

In addition to IMT, our patient presented with extensive pelvic retroperitoneal fibrosis that extended into the seminal vesicles and Denonvilliers’ fascia and encased the inferior ureter. The inferior ureters were soft and appeared normal following the removal of their fibrotic shell. Partial cystectomy was carried out through intraoperative freezing, and nearly the entire bladder was removed, leaving only tissue normal in appearance. In our patient, the prostate was not taken away, and the neurovascular bundle was not spared due to the fibrotic lesion of bladder. Although the patient showed no evidence of IMT recurrence and no erectile dysfunction, close follow-up is necessary.

## Conclusion

The IMT of the 17-year-old boy in this case involved the entire bladder and showed a tendency for invasive growth, although it affected mainly adjacent fixed tissue. The clinical, radiographic and pathological characteristics of the tumor were between those of benign and malignant lesions. The patient presented with elevated levels of inflammatory response markers, which fell after surgery. The patient also presented with extensive pelvic retroperitoneal fibrosis involving the entire bladder and several adjacent pelviabdominal organs. The bladder wall was tough and could hardly be cut by scalpel. Close follow-up is necessary to check for tumor recurrence or metastasis.

## Consent

Written informed consent was obtained from the patient for publication of this case report and accompanying images. A copy of the written consent is available for review by the Editor-in-Chief of this journal.

## Abbreviations

AFP: α-fetoprotein; ALK: Anaplastic lymphoma kinase; CEA: Carcinoembryonic antigen; CT: Computed tomography; IMT: Inflammatory myofibroblastic tumor; NVB: Neurovascular bundle.

## Competing interests

The authors declare that they have no competing interests.

## Authors’ contributions

XLY, HYL and YXW contributed equally to this work, participated in the design of the study, performed the clinical analysis and drafted the manuscript. WHL, QGM, JWC, YT participated in study design, literature search and coordination. XLY, HYL, YL and XZB performed the operation. All authors participated in the
